# Defined Nutrient Diets Alter Susceptibility to *Clostridium difficile* Associated Disease in a Murine Model

**DOI:** 10.1371/journal.pone.0131829

**Published:** 2015-07-16

**Authors:** John H. Moore, Caio C. D. Pinheiro, Edna I. Zaenker, David T. Bolick, Glynis L. Kolling, Edward van Opstal, Francisco J. D. Noronha, Pedro H. Q. S. De Medeiros, Raphael S. Rodriguez, Aldo A. Lima, Richard L. Guerrant, Cirle A. Warren

**Affiliations:** 1 Division of Infectious Diseases and International Health, University of Virginia, Charlottesville, Virginia, United States of America; 2 Biomedicine Institute, Federal University of Ceará, Fortaleza, Brazil; Charité, Campus Benjamin Franklin, GERMANY

## Abstract

**Background:**

*Clostridium difficile* is a major identifiable and treatable cause of antibiotic-associated diarrhea. Poor nutritional status contributes to mortality through weakened host defenses against various pathogens. The primary goal of this study was to assess the contribution of a reduced protein diet to the outcomes of *C*. *difficile* infection in a murine model.

**Methods:**

C57BL/6 mice were fed a traditional house chow or a defined diet with either 20% protein or 2% protein and infected with *C*. *difficile* strain VPI10463. Animals were monitored for disease severity, clostridial shedding and fecal toxin levels. Select intestinal microbiota were measured in stool and *C*. *difficile *growth and toxin production were quantified *ex vivo *in intestinal contents from untreated or antibiotic-treated mice fed with the different diets.

**Results:**

*C*. *difficile *infected mice fed with defined diets, particularly (and unexpectedly) with protein deficient diet, had increased survival, decreased weight loss, and decreased overall disease severity. *C*. *difficile* shedding and toxin in the stool of the traditional diet group was increased compared with either defined diet 1 day post infection. Mice fed with traditional diet had an increased intestinal Firmicutes to Bacteroidetes ratio following antibiotic exposure compared with either a 2% or 20% protein defined nutrient diet. *Ex vivo* inoculation of cecal contents from antibiotic-treated mice showed decreased toxin production and *C*. *difficile* growth in both defined diets compared with a traditional diet.

**Conclusions:**

Low protein diets, and defined nutrient diets in general, were found to be protective against CDI in mice. Associated diet-induced alterations in intestinal microbiota may influence colonization resistance and clostridial toxin production in a defined nutrient diet compared to a traditional diet, leading to increased survival. However, mechanisms which led to survival differences between 2% and 20% protein defined nutrient diets need to be further elucidated.

## Introduction

*Clostridium difficile* is an anaerobic, spore-forming, gram-positive bacterium [1] that causes disease mainly through the activity of two toxins, TcdA and TcdB. Some *C*. *difficile* strains produce a third toxin, binary toxin (CDT) or *C*. *difficile* transferase, that may contribute to hypervirulence (such as in the strain Nap1/ 027) [2]. *C*. *difficile* is also recognized as the major identifiable and treatable causative agent of antibiotic-associated diarrhea[3]. *C*. *difficile* infection (CDI) has a wide clinical range from asymptomatic carriage to mild self-limiting diarrhea to severe pseudomembranous colitis (PMC) [4]. With the emergence of hypervirulent strains and increased use of antibiotics incidence of CDI has dramatically increased during recent years. Studies performed in Canada, USA and Europe report increases as much as two to four fold in CDI incidence in the past decade [5, 6].

Antibiotic exposure is the most important risk factor for developing CDI [7]. Before the advent of antimicrobial agents, PMC was a relatively rare disease, largely associated with colonic, pelvic, or gastric surgeries [4]. Commensal microbiota inhibit colonization and overgrowth of *C*. *difficile* and other enteric pathogens [8, 9]. Altered intestinal microbiota composition was observed in patients with antibiotic-associated diarrhea [10]. Besides the use of antibiotics there are also several other important risk factors associated with CDI including: old age [7, 11], frequent hospitalizations, and prolonged hospital stays [12, 13]. The effect of nutritional status on CDI has not been investigated.

Poor nutritional status is well recognized as a widespread health problem with consequences that are both acute and often long-term. Malnutrition contributes to mortality, directly or indirectly, through weakened defenses against other diseases such as malaria, respiratory, or diarrheal diseases [14]. It has been shown that for some enteric infections, such as cryptosporidiosis, giardiasis, and enteroaggregative *E*. *coli*, a protein deficient diet results in worse outcomes from these diseases in murine models of enteric infection [15–17].

Diet alters the intestinal microbiota in humans [18]. Specific nutrients can be associated with gut microbiota changes that alter severity of gut inflammation (*e*.*g*. *Bilophilia wadsworthia* overgrowth from excess saturated fat consumption in IL-10 knockout mice leading to worse chronic colitis [19] which normally occurs in this animal model), while probiotics (a combination of *Lactobacillus*, *Bifidobacterium* and *Streptococcus salivarius*) may help relieve Inflammatory Bowel Disease in humans [20]. Since diet alters gut microbiota and nutritional status, including protein deficiency, affects susceptibility to infection with other enteric infections, we examined how a protein malnourished diet may alter severity of disease in a murine model of CDI, and examined the role of a reduced protein diet on selected commensal microbiota and burden of infection in a murine model. Surprisingly, we found protein a protein malnourished diet conferred protection against mortality from CDI, while a traditional diet had increased mortality compared with either defined nutrient diet, as well as associated increases in pathogen burden and microbiota changes.

## Methods and Materials

### Mouse Diets

**Fig 1:**
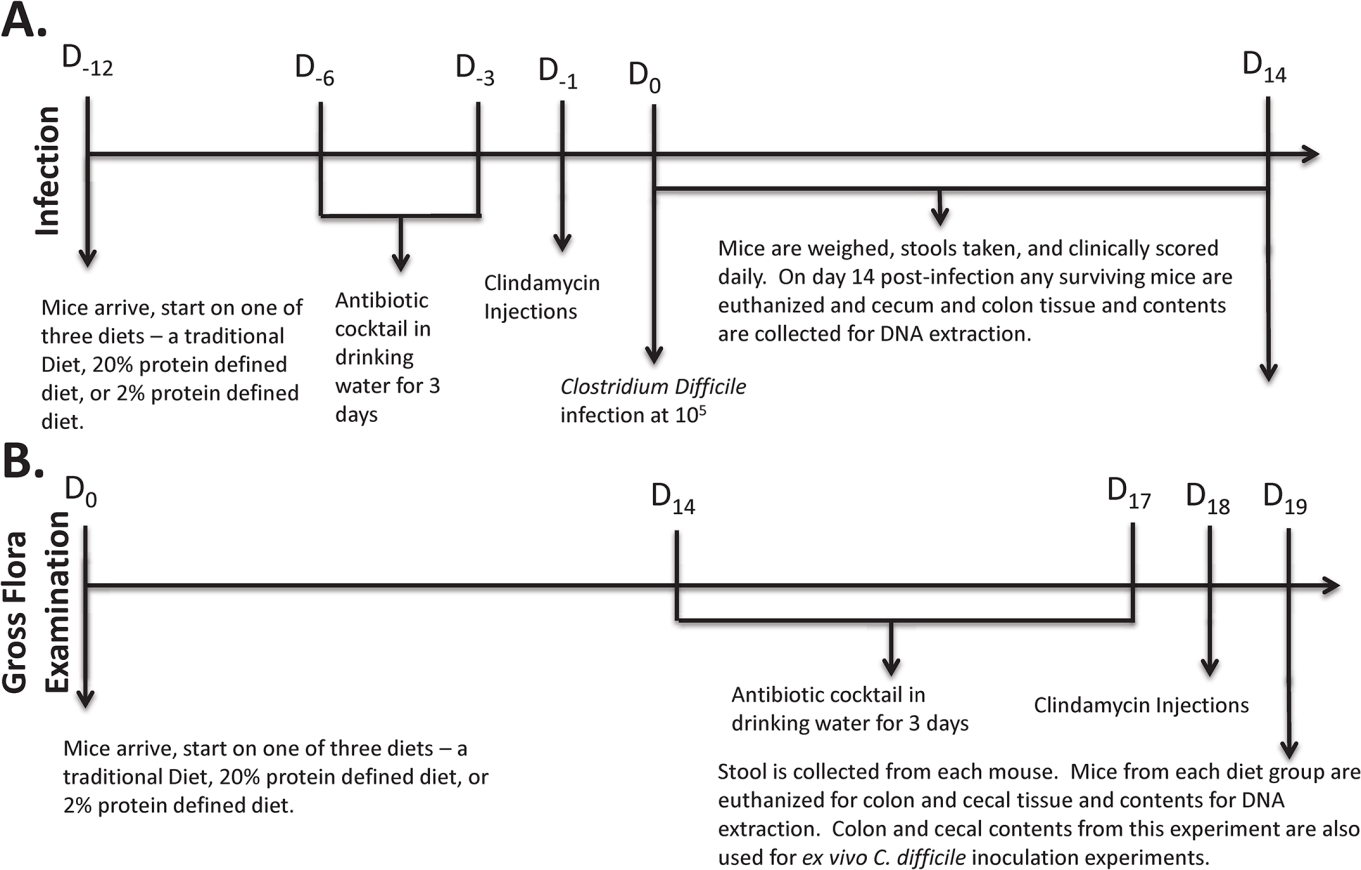
Treatment schedule for mice for the *in vivo* infection model (A) and *ex vivo C*. *difficile* inoculation studies (B).

To assess different nutritional status in mice, five separate diets were used: a protein malnourished diet with 2% protein by weight (Harlan Labs, Madison, WI, number TD.08679) and its matched nourished control diet with 20% protein by weight (Harlan Labs, Madison, WI, number TD.08678), a regional basic malnutrition diet (Research Diets, number D09051102) and its matched control (Research Diets, number D09081701) [21], and a natural ingredient “traditional” house chow diet (Harlan Labs, Madison, WI, Teklad number 7012). The macronutrient composition of the diets is shown in **Table 1**(diet ingredient compositions in **S1, S2, S3, and S4 files**). The *traditional* diet is a diet where ingredients were not composed of a single macronutrient and included ingredients such as corn, wheat and soybean meal, this diet is used in our other models of fulminant CDI and will be used in this experiment as positive control for infection [22, 23]. The *defined* diets (all diets used aside from the traditional diet) are composed of a known macronutrient such as protein from casein or carbohydrates from starch. All diets were irradiated at the source company prior to shipping. The mice were started on the protein deficient diets and their matched controls 12–14 days prior to infection in the infection model (**Fig 1A)** or 19 days prior to euthanasia in the gut microbiota and ex-vivo studies (**Fig 1B**). All diets used were isocaloric. Food and water were given ad libitum to the mice. Weights and clinical scoring were recorded daily post-infection.

**Table 1:** Macronutrient Composition of Diets Used.

Nutrient		Traditional Diet	20% Protein Defined Diet	2% Protein Defined Diet	Regional Basic Control Diet	Regional Basic Diet
**Protein**	% weight	19.1	20.3	2	19	6.5
	% by kcal	25	22.3	2	20	7
**Carbohydrate**	% weight	44.3	58.3	76.8	63.1	81.9
	% by kcal	58	64.1	84.2	65	88
**Fat**	% weight	5.8	5.5	5.5	6.5	2.1
**Energy Density**	% by kcal	17	13.6	13.6	15	5
	kcal/g	3.1	3.6	3.6	3.7	3.7

### Murine model of *C*. *difficile* infection

The infection model is a modified version of the published protocol by *Chen et al* [24, 25]. This protocol has been approved by the Center for Comparative Medicine at University of Virginia (Protocol #3626). C57BL/6 mice (Jackson Laboratory, Bar Harbor, ME), male, between 7–8 weeks old on arrival were used in all studies. Mice were randomly assigned treatment groups prior to the experiment, and singly housed immediately following infection. As shown in **Fig 1A**, starting 6 days prior to infection, mice were given an antibiotic cocktail containing vancomycin (0.0045mg/g), colistin (0.0042mg/g), gentamicin (0.0035mg/g), and metronidazole (0.0215mg/g), which was discontinued 3 days prior to infection. One day prior to infection, clindamycin (32mg/kg) was injected intraperitoneally. All antibiotics were obtained from the University of Virginia Hospital Pharmacy. Infection was performed with live, vegetative *C*. *difficile* strain VPI10463 (ATCC) at 10^5^ CFU by oral gavage. Daily weight changes, diarrhea and disease severity scores were recorded as previously described [22] and are based on weight change from day 0 (0–4 points), posture (0–3), coat appearance (0–3), diarrhea (0–3), eye appearance (0–3), and activity (0–4). Stool samples were collected at select timepoints. Moribund mice, mice with clinical scoring > 14, or mice that had lost more than 25% of their infection day bodyweight were euthanized with a ketamine/xylazine cocktail administered via intraperitoneal injection followed by manual cervical dislocation. All surviving mice at 2 weeks post-infection were euthanized for sample collection (intestinal contents and tissue). Stool and cecal contents samples were stored at -20°C until processed for toxins A/B and bacterial quantification assays. Cecal and colonic tissue samples were fixed in 10% zinc formalin, paraffin embedded, and stained using hematoxylin/eosin (services provided by University of Virginia Research Histology Core). A blinded reader scored the coded slides using published parameters [23].

### Toxin A/B assay

To determine toxin load in stool a *C*. *DIFFICILE TOX A/B II* test (Techlab, Blacksburg, VA) was used and samples were processed as recommended by the manufacturer. Samples were normalized according to weights using diluent provided in the kit. Samples were diluted 10x and optical density (OD) readings were taken at 450/620 nm (readings taken at 450 nm, blanked against air at 620 nm).

### Bacterial quantification assays

DNA was extracted from the thawed samples (stool and cecal contents) using the QIAamp DNA Stool Kit (Qiagen, Valencia, CA) with minor modifications; stool samples were suspended in 200 ul diluent from the Toxin A/B assay kit and half was taken for toxin analysis prior to processing. All qPCR experiments were performed in the CFX96 Real-Time PCR Detection System (Bio-Rad Laboratories, Inc., Hercules, CA). A total reaction volume of 20 μl per sample was prepared by mixing 10 μL of Quantifast SYBR Green Supermix (Qiagen), 1 μL of each primer (Eurofins MWG Operon, Huntsville, AL) at 10 μM (final concentration 0.4 μmol/L), 4 μL of template DNA and 4 μL of DEPC-treated nuclease free sterile water (Fisher Scientific, Pittsburgh, PA). The primers used for DNA quantification were as follows: *tcdB* foward primer (5’- GGTATTACCTAATGCTCCAAATAG—3’) and *tcdB* reverse primer (5’- TTTGTGCCATCATTTTCTAAGC—3’) were used for *C*. *difficile* detection [26]; 16S forward primer (5’- GGCTAACTCCGTGCCAGCA-3’) and 16S reverse primer (5’-GCGCTTTACGCCCAGTAATTC—3’) were used for universal bacterial detection; Bact934 forward primer (5’- GGARCATGTGGTTTAATTCGATGAT -3’) and Bact106 reverse primer (5’- AGCTGACGACAACCATGCAG—3’) were used for Bacteroidetes detection; Firm350 forward primer (5’—GGCAGCAGTRGGGAATCTTC—3’) and Firm814 reverse primer (5’—ACACYTAGYACTCATCGTT—3’) were used for Firmicutes detection; and Uni515 forward primer (5’—GTGCCAGCMGCCGCGGTAA—3’) and Ent826 reverse primer (5’–GCCTCAAGGGCACAACCTCCAAG—3’) for Enterobacteriaceae detection (16s, Bacteroidetes, Firmicutes, and Enterobacteriaceae primers were from [27]). The 2-step amplification reaction consisted of 95°C for 5 min, then 40 cycles of a denaturing step at 95°C for 10 s, and an annealing/extension step at (57°C for *C*. *difficile* and 59°C for Bacteroidetes, total bacteria, and Firmicutes) for 30 s. Following amplification, melting curve analysis was carried out by 0.5-degree increments for 5s starting at 65°C and ending with 95°C to determine the specificity of PCR reactions. The fluorescence signal was measured during the annealing step of each cycle and the Ct values of each run were compared to standards with known concentrations of bacterial DNA. The final DNA values were normalized to the samples’ initial stool weight.

### Examination of the effects of diets on selected microbiota in mice

To examine changes in microbiota from different diets and antibiotics, mice were placed on different diets (Harlan Traditional diet (catalogue number 7912), Harlan 2% protein defined diet (TD.08679), Harlan 20% protein defined diet (TD.08678)) for 19 days. Select groups were also given antibiotics 14 days after starting the diets as described in the *ex vivo* inoculation studies (**Fig 1B)**. After 19 days mice were euthanized and both colon and cecum contents and tissue were collected for DNA extraction and qPCR, as described in the Bacterial Quantification Assays section. Colon and cecum tissue sections were washed in phosphate buffered saline to remove any contents adhered before further analysis.

### Murine model of colonization resistance to *C*. *difficile*

This published model [28] was modified for this study. Mice were given respective diets and pretreated with antibiotics as described above. Following antibiotic treatment (**Fig 1B**), mice were euthanized and cecal contents were collected and stored at -20°C until processed. Under anaerobic conditions, cecal contents were diluted threefold in sterile pre-reduced phosphate-buffered saline (PBS), inoculated with 10^4^ CFU *C*. *difficile* strain VPI 10463, and incubated for 24 hours. Clostridial and toxin levels were measured by PCR and ELISA, respectively, as described above.

### Statistical methods

Statistical analyses were performed using GraphPad Prism version 5.0 (GraphPad software, San Diego, CA). Data was presented as mean +/- standard error of the mean (SEM). Significance in all cases was set at p≤0.05. Survival data was analyzed by Log-rank (Mantel-Cox) survival analysis. Scores, weights, and assay values were analyzed using a One-way, Two-way, or Repeated Measures Analysis of Variance (ANOVA) test, or student’s two-tailed t-test where appropriate. A Bonferroni correction post-test was used in conjunction with significant ANOVA results to determine where significant differences between groups lay. With bacterial quantification assays, the log 10 values of the results were used to achieve normal distribution.

## Results

### A Protein Deficient Diet or Defined Diet is protective against severe *C*. *difficile* infection

**Fig 2:**
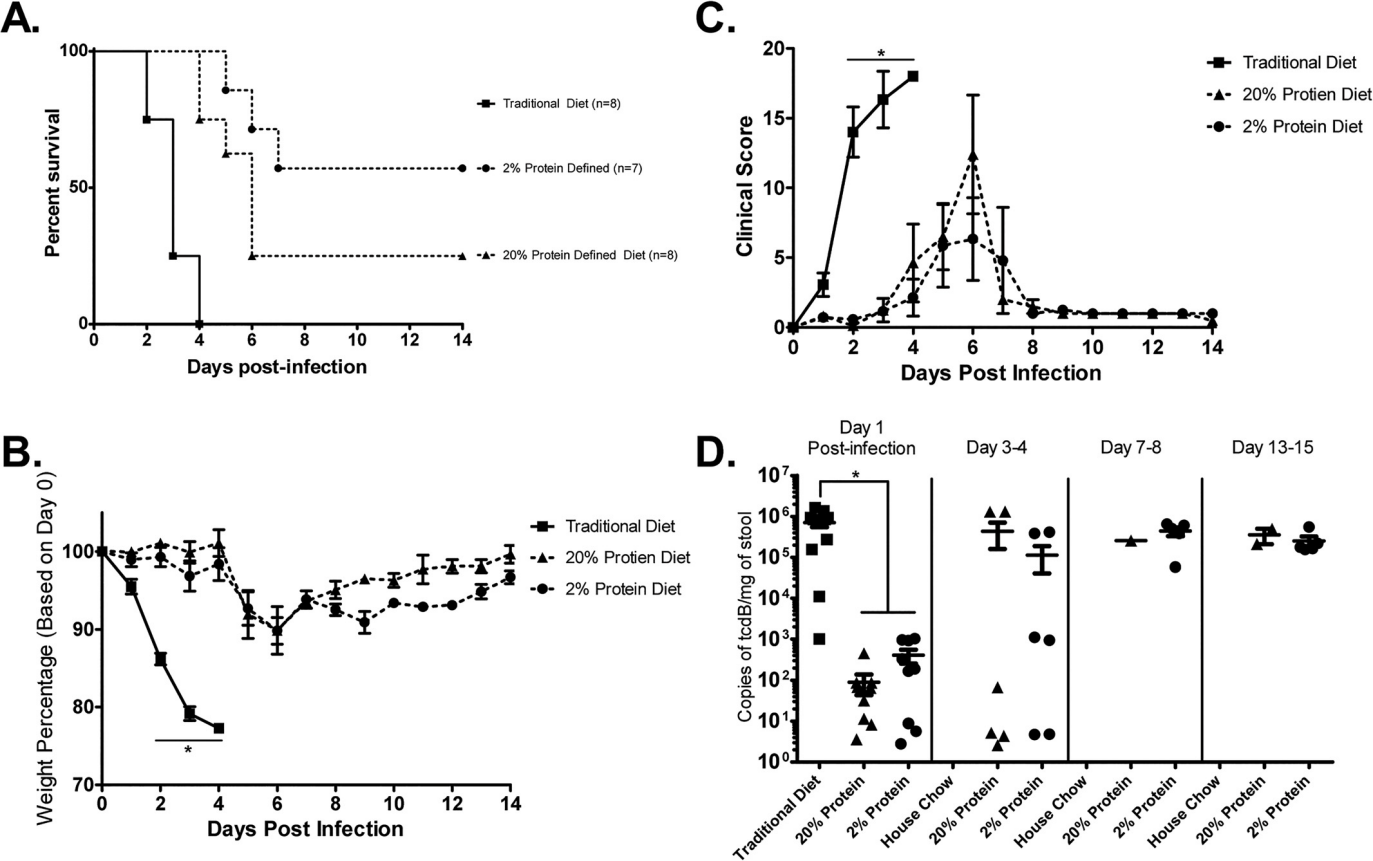
Effects of nutritional status and diets on mortality, disease severity and infection burden in mice challenged with *C*. *difficile*.

To determine the effect of a malnourishing diet to susceptibility to CDI, mice were fed with either a traditional diet, a 20% protein defined nutrient diet, or a 2% protein defined nutrient diet. After inoculation with *C*. *difficile* strain VPI 10463, mice fed the traditional diet progressively developed diarrhea and weight loss **(Fig 2)**. All mice in this group succumbed to infection by day 4 post-infection (0/8). Mice on the 20% protein diet demonstrated delayed onset of disease and overall 14 day post-infection survival of 25% (2/8), while mice on the 2% protein diet also had delayed onset of disease and had a survival rate of 57.1% (4/7). Log-rank tests confirmed significant differences in survival curves between the traditional and 20% protein diets, the traditional and 2% protein diets, but not between the 20% and 2% protein diets. These findings were replicated in another experiment comparing infected mice fed with our regional basic malnutrition diet (6.5% protein) and its control (19.5% protein) with the traditional diet (**S1 Fig**). Mice on the defined Regional-Basic Diet (RBD) and matched control diets had lower mortality rates (7 day post-infection survival of 100% (8/8) and 87.5% (7/8), respectively) compared with the traditional diet (0% survival, (0/4)). Fecal *C*. *difficile* levels were elevated in stool from traditional diet fed mice on day 1 compared with mice on other diets **(Fig 2D)**. *C*. *difficile* shedding increased from day 1 to day 3–4 post infection in surviving mice from the 20% and 2% protein diet groups. Similarly, *C*. *difficile* toxin A/B load in stool was significantly greater in the traditional diet receiving group day 1 post-infection than either the 20% or 2% protein defined diet groups **(S2 Fig)**. Interestingly, although *C*. *difficile* fecal burdens were persistently elevated in surviving mice, toxin load was variable in the defined diet groups from day 3–4 post-infection onwards. Overall, animals receiving a defined nutrient diet had delayed onset of disease, increased survival, and initial decreased pathogen shedding and fecal toxin production compared with a traditional diet. Animals fed a reduced protein diet had decreased mortality compared to their matched control (20% defined nutrient protein diet), although this difference was not statistically significant.

Mice were on indicated diets starting 12–14 days prior to and 14 days post-infection with *C*. *difficile* strain VPI 10463.**A: Survival rate.** Log-Rank tests confirmed significant differences between the traditional diet and each the 20% and 2% protein diets (p<0.05) but not a significant difference between the 20% and 2% protein diet groups. **B: Weight Change.** Significant differences (p < 0.0001) in weight percentages between the traditional diet and the 20% and 2% protein diets were seen on days 2–4 post-infection. **C: Clinical Score.** Significant (p < 0.01) differences on days 2, 3 and 4 post-infection were seen between the traditional diet and the 20% and 2% protein diets; but there was no significant difference in clinical scoring between the 20% and 2% protein diets. **D: Fecal *C*. *difficile* Shedding qPCR**. *C*. *difficile* shedding significantly increased in the traditional diet receiving group compared with either the 20% or 2% protein diets at day 1 post-infection. Stool was not available from the traditional diet group after day 1 as the mice were either too sick to give stool or deceased. There weren’t significant differences between 20% and 2% organism shedding at each timepoint, but there were increases in *C*. *difficile* shedding in each of the 20% and 2% protein diet groups from day 1 to day 3–4 post-infection (p<0.001).

### Antibiotic-induced alteration in intestinal microbiota composition is influenced by diet

Intestinal dysbiosis, either through previous antibiotic exposure, radiation, or chemotherapeutic agents influences susceptibility to CDI. Therefore, we interrogated whether the decreased susceptibility to severe disease from CDI was associated with diet-induced changes in intestinal microbiota in the murine model which could help explain survival differences between diet receiving groups. We chose four groups of gut microbiota to survey in the experiment: Firmicutes (Phyla level), Bacteroides (Phyla level), Enterobacteriaceae (Family level), and total bacteria. These phyla were chosen as they represent the majority of bacterial species in the mouse gastrointestinal microbiota [27, 29].

**Fig 3:**
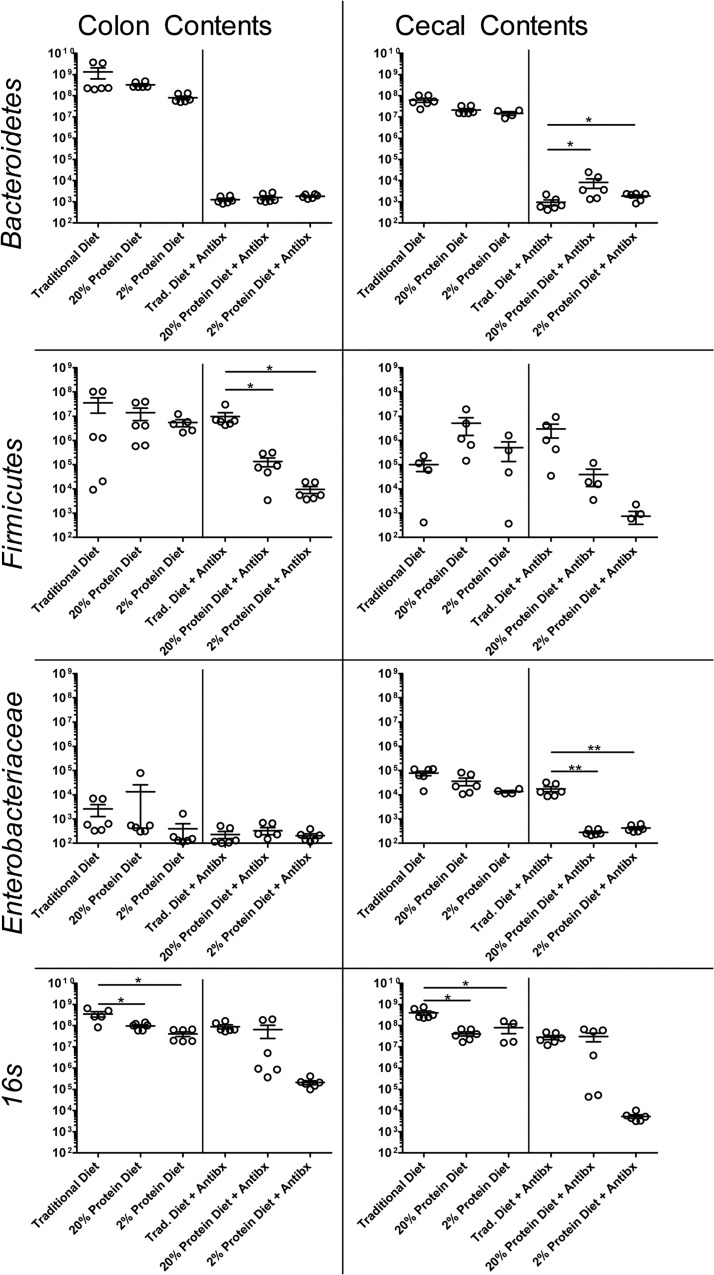
Quantification of resident gut microbiota phyla *Firmicutes*, *Bacteroidetes*, and *Enterobacteriaceae*, and total bacteria using qPCR. Intestinal contents were taken from mice fed either a traditional, 20% defined, or 2% defined diet as outlined in **Fig 1B.** Mice fed with indicated diets were treated with and without antibiotics. N = 3 animals per group, with each sample run in duplicate and results normalized to 10μg of sample. *Bacteroidetes* was significantly higher in the cecal contents of mice receiving either a 2% or 20% defined diet compared with traditional diet (p<0.05) with antibiotics, *Firmicutes* in colon contents was significantly higher in the traditional diet than either defined diet with antibiotics (p<0.05), *Enterobacteriaceae* was significantly higher in the cecal contents of mice fed a Traditional diet compared with either defined diet (p<0.001), and total bacteria was significantly higher in both colon and cecum contents of mice fed a traditional diet versus either defined diet.

**Fig 4:**
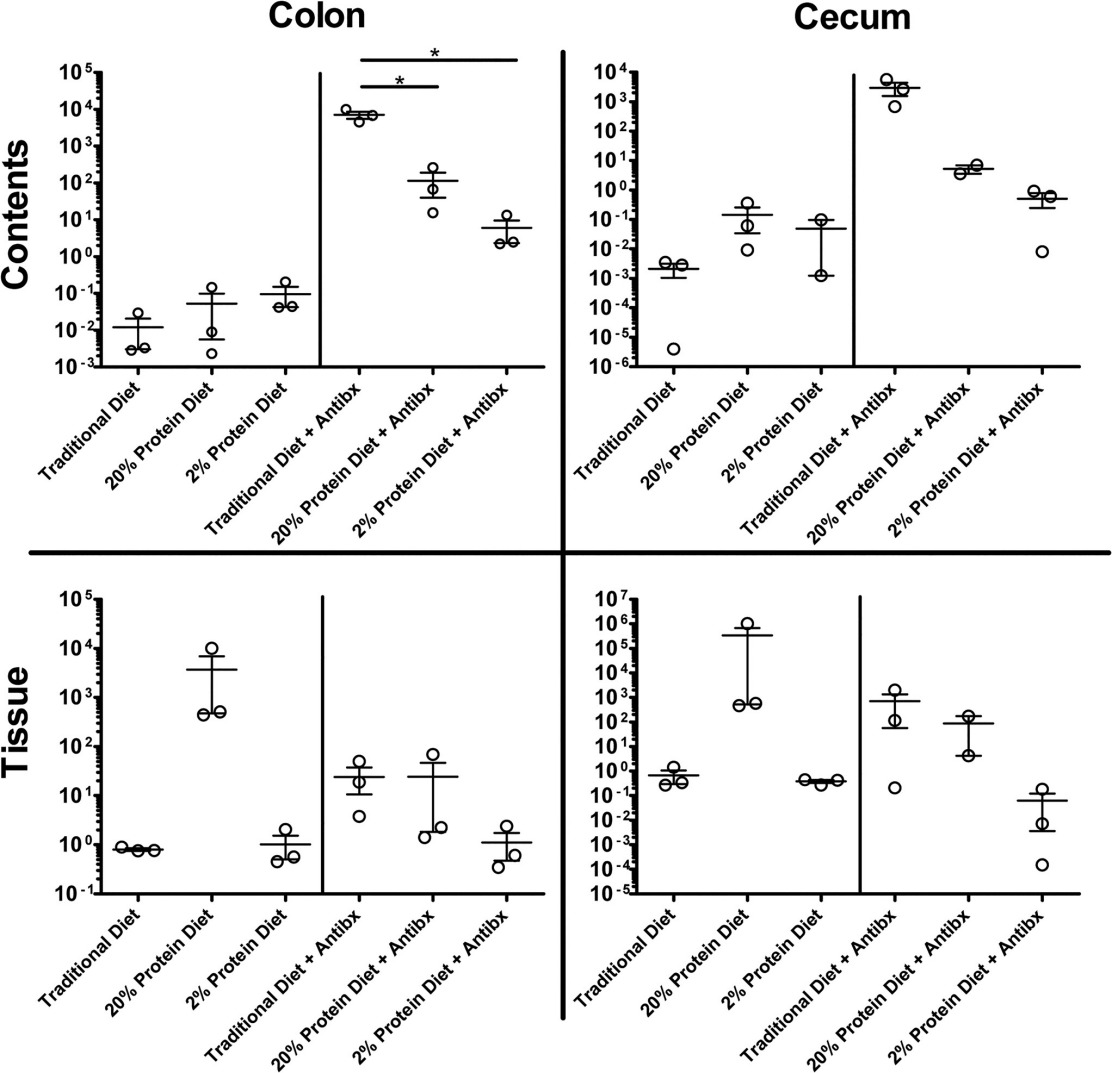
*Firmicutes* to *Bacteroidetes* ratio. *Firmicutes* to *Bacteroidetes* ratio in the colon contents (lumen) was significantly higher (ANOVA p = 0.0021, post-tests both p<0.01) in the traditional diet fed group treated with antibiotics compared with either defined nutrient diet with antibiotics.

The experimental design is shown in **Fig 1B**, with N = 3 for each experimental group. In the absence of antibiotics, the total bacterial (16s) burden was significantly elevated in colon and cecum contents from mice on traditional diet compared with either defined diet (p<0.05) and select intestinal microbiota- Bacteriodetes, Firmicutes and Enterobacteriaceae, were not significantly different among the groups **(Fig 3)**. However, among mice treated with antibiotics, Bacteroidetes was significantly more depressed in the cecal contents of those receiving a traditional diet compared with either defined diet (p<0.05). In contrast, Firmicutes in colon contents and Enterobacteriaceae in cecum contents from antibiotic-treated mice on traditional diet were significantly higher compared with either defined diet (p<0.05 in both cases). The ratio of Firmicutes to Bacteroidetes is another measure of microbiome composition. Age, obesity, and antibiotic usage are factors which have been shown to affect this ratio [30–32]. We found that the Firmicutes to Bacteroidetes ratio in the colon contents was significantly higher in the group receiving traditional diet with antibiotics compared with either defined diet receiving group with antibiotics **(Fig 4)**. This finding is consistent with what was previously reported that a higher Firmicutes to Bacteroidete*s* ratio was associated with risk of acquiring CDI [33]. Changes in select bacterial microbiota from intestinal tissues were not as consistent but are presented in **S3 Fig.**

### Cecal contents from mice fed a traditional diet allow for increased growth of *C*. *difficile* bacteria *ex-vivo*

**Fig 5:**
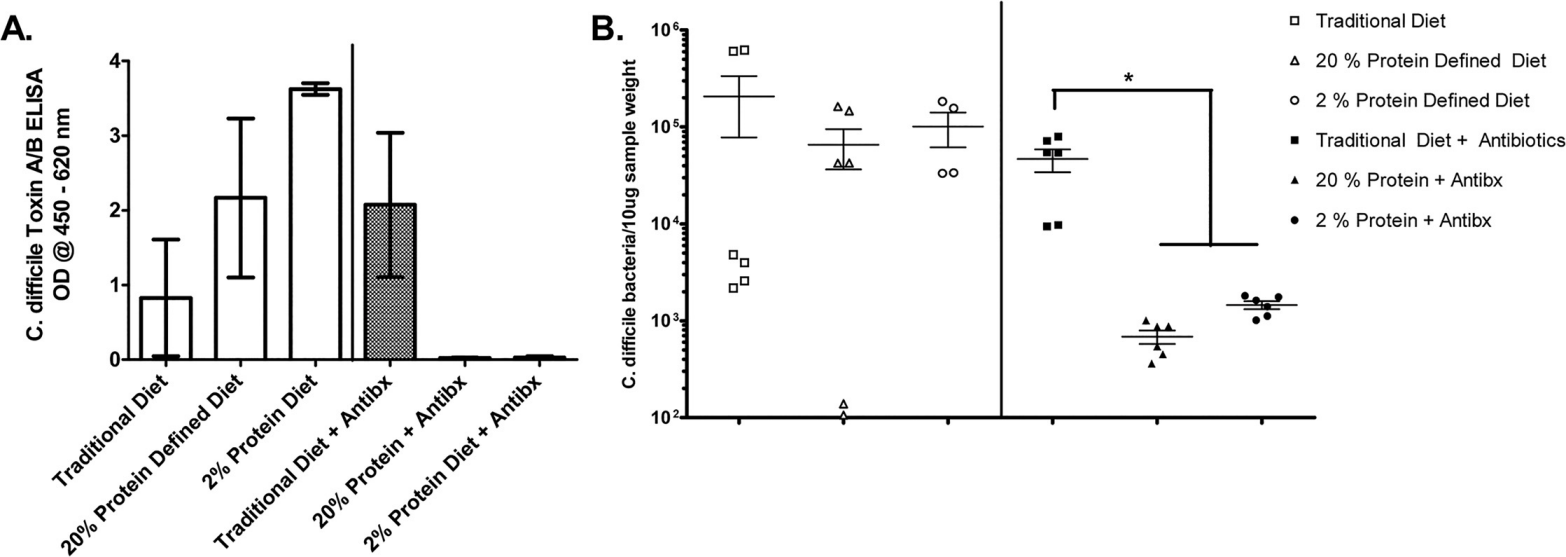
*Ex vivo* Inoculation of Cecum Contents with *C*. *difficile*. **A.** *C*. *difficile* toxin levels (Toxin A/B ELISA) in cecal contents 24 hours post-inoculation with 10^4^ VPI 10463 (N = 3 for each group). Oneway ANOVA with Bonferroni correction post-test did not show significant differences between groups **B.** Quantitative Real-Time PCR of *C*. *difficile* Toxin B gene, *tcdB*, in inoculated cecal contents 24 hours post-inoculation (N = 3 for each group, results in duplicate). There was significantly higher *C*. *difficile* growth in the traditional diet cecal contents with antibiotics compared with either the 20% diet group with antibiotics (p < 0.01) or with cecal contents from the 2% protein diet group with antibiotics (p < 0.01). No significant differences were detected between groups not receiving antibiotics

To examine diet and antibiotic-induced alteration of the intestinal milieu to the cecal contents’ conduciveness to clostridial growth and toxin production cecal contents were inoculated with *C*. *difficile* bacteria and incubated for 24 hours (N = 3 per group). Growth of *C*. *difficile* was not different in cecal contents across all diet groups not exposed to antibiotics (**Fig 5A)**. However with antibiotic exposure inoculation of cecal contents from mice fed the traditional diet yielded significantly higher clostridial loads compared to cecal contents from the 20% diet group (p < 0.01) or from the 2% protein diet group (p < 0.01). Likewise, *C*. *difficile* toxin levels were lower in the cecal contents from defined diet groups treated with antibiotics compared with traditional diet with antibiotics but the difference was not statistically significantly (**Fig 5B)**. These findings suggest that antibiotics differentially alter the gut microenvironment on mice fed different diets (thus, unmasking diet effects on the microbiota), leading to changes in *C*. *difficile* growth and toxin production following inoculation.

## Discussion

In the mouse model we found that different diets influenced susceptibility to CDI, and in the presence of antibiotics, altered select intestinal microbiota as well as *C*. *difficile* growth and toxin production. Contrary to our initial hypothesis, malnutrition using defined protein deficient diets did not increase susceptibility to CDI and was actually protective against severe disease. In contrast to CDI, enteric infections common in resource-limited settings such as cryptosporidiosis, giardiasis, and enteroaggregative *E*. *coli* infection were previously reported to be exacerbated by malnutrition induced by a low protein diet in animal models [15–17]. These findings may help explain differences in prevalence of CDI between resource-sufficient and resource-limited settings.

The traditional diet conferred increased susceptibility to infection compared to the defined nutrient diets. One of the primary differences in the diet compositions is the abundance of fermentable fiber in the traditional diet compared with the defined nutrient diet, which had none. The traditional diet contains wheat middlings, which contain up to 100% fructo-oligosaccharides (FOS) [34]. The defined diets contain only cellulose, a non-fermentable ingredient as fiber source. Other carbohydrates added to the defined diets include sucrose, maltodextrin, and corn starch; all of which are readily absorbed before they reach the colon. FOS and other fermentable fibers are not digestible in mammals but are fermented in the colon by anaerobic bacteria which can cleave their (1–2) beta glyosidic bonds. These promote the growth of resident anaerobic bacteria including *Lactobacillus*, *Bifidobacterium*, and *Clostridium* species, which may enhance colonization resistance against CDI. With broad-spectrum antibiotic treatment, these beneficial microbiota are decreased and FOS is left available for toxigenic *C*. *difficile*. Indeed, in a study where stool taken from healthy volunteers was inoculated with *C*. *difficile*, colonization resistance conferred by supplementation of fecal slurries with FOS was lost in the presence of clindamycin [35]. With clindamycin, *C*. *difficile* growth increased and bifidobacterial counts decreased in stool supplemented with FOS compared with stool exposed to clindamycin without FOS. These findings suggest that the effect of clindamycin on *Bifidobacterium* (and other potentially beneficial anaerobes) resulted in an environment which suppressed beneficial microbiota but left more fermentable fiber nutrient substrate for the *C*. *difficile* bacteria. Therefore, we hypothesize that while FOS and fermentable fiber can help resolve large bowel dysbiosis, they can also feed and stimulate pathogenic *C*. *difficile* bacteria already present in the gut, potentially aggravating the disease. Consistent with the previous study, ex vivo inoculation of cecal contents from antibiotic-treated mice, in our study, showed decreased toxin production (though not statistically significant) and significantly decreased *C*. *difficile* growth in both defined diets compared with the FOS-rich traditional diet. However, FOS supplementation was also shown by others to increase fecal concentration of *C*. *difficile* in antibiotic-treated mice but decreased fecal toxin A titers and modulated inflammatory response to the infection [36]. These observations suggest that a component of the intestinal compartment environment, likely microbiota as influenced by the diet, may influence *C*. *difficile* growth, toxin production, and therefore, outcome of infection. Indeed, in humans, diet may alter host response to disease by its effects on the gut microbial composition [37]. More studies are needed to elucidate how diet affects response to CDI and the potential benefit of dietary manipulation for prevention or treatment of infection.

Of note, we have also observed that infected mice fed protein deficient defined diet had better survival although not significantly compared to defined diet control. *C*. *difficile* ferment protein as a food source in the absence of fermentable oligosaccharides [38], Thus, limited alternative energy or nutrient source for *C*. *difficile* may help explain some differences in outcome between the 20% and 2% defined protein diet groups. However both *in vivo* and *ex vivo* experiments showed no difference in *C*. *difficile* burden among mice fed low vs sufficient protein diets. The mechanism by which differences in outcome occur between 2% and 20% protein defined diets needs to be further elucidated.

Disruption of the intestinal microbial community by antibiotics is an overarching risk factor for *C*. *difficile* colonization and infection [39]. We found that gut microbiota composition was different between traditional and defined nutrient diets in the presence of antibiotics. Although microbial changes varied among the source of DNA material—i.e. cecal vs colonic, tissue vs luminal contents, the bacterial groups that appeared to be most affected were Firmicutes and Bacteroidetes. Firmicutes were significantly higher in colon contents of mice fed a traditional diet compared with either defined diet after antibiotic treatment. While colonization with certain clostridial groups and species had been reported to be protective against subsequent colonization or infection with pathogenic *C*. *difficile*, *C*. *difficile* itself, is a member of the Firmicutes phylum, suggesting that the nutritive conditions following antibiotic treatment in mice fed traditional diet were such that clostridial growth was favored. Indeed, we confirmed with our *ex vivo* experiments that *C*. *difficile* growth was significantly higher in cecal contents of animals receiving a traditional diet post-antibiotic administration compared with either defined diet after receiving antibiotics. In addition, Bacteroidetes was relatively preserved in cecal contents from mice fed with defined diets with antibiotic exposure, likely contributing to the decreased susceptibility of these mice to CDI (27). Microbial population dynamics also changed, as the Firmicutes to Bacteroidetes ratio was significantly higher in colon contents of antibiotic treated mice fed a traditional diet compared with either defined nutrient diet. In human disease, increased Firmicutes to Bacteroidetes ratios have been shown to be a marker of susceptibility to acquisition of CDI [33].

Limitations in the scope of this study include lack of analysis of immune system function between a 2% and 20% protein defined diet. The direct effects of diet on immune cells have been previously recognized but these effects are closely intertwined with the profound influence of diet to the commensal microbiota as well [40]. Intestinal dysbiosis is the critical factor in the development of CDI, however, the balance of host anti- and pro-inflammatory responses may define disease severity [41]. In addition, deficiency of key micronutrients such as zinc have been shown in murine models to both upregulate virulence factors of the pathogen and increase the pro-inflammatory response of the host, leading to worse outcomes [42]. Moreover, a more comprehensive microbiome analysis may further detect alterations specific to various diets. The dynamic interaction of diet, immune response, microbiota and pathogen warrants further studies.

In conclusion, diet plays a role in susceptibility and outcomes of CDI. Examining the impact of specific ingredients or nutrients (especially FOS) to intestinal microbiota, colonization resistance, clostridial growth and toxin production, and immune response, may lead to a better understanding of the pathogenesis and development of alternative preventive measures and new therapies against CDI.

## Supporting Information

S1 Fig Effects of nutritional status and Regional-Based diet on mortality, disease severity, and infection in mice challenged with *C*. *difficile*. Mice were on indicated diets starting 12 days prior to and 7 days post-infection with *C*. *difficile* (VPI 10463) at 10^5^ CFU inoculum. A, Survival Rate. B, Clinical Scoring. C, Weight Change. D, Histopathology Scores.
(EPS)

S2 Fig ELISA for *Clostridium difficile* toxins A/B. *C*. *difficile* toxin A/B load in stool was significantly greater in the traditional diet receiving group day 1 post-infection (O.D. = 3.681 ±0.201 SEM) (one-way ANOVA with Bonferroni post-test; p<0.05 between traditional diet receiving group and either the 20% (O.D. = 0.003±0.001 SEM) or 2% (O.D. = 0.004 ±0.0003 SEM) defined protein diet group.
(EPS)

S3 Fig Quantitative-real time PCR of genomic DNA for resident gut microbiota in intestinal tissues. *Bacteroidetes* was significantly higher in colon mucosa of mice receiving the traditional diet compared with either defined nutrient diet (p<0.05), but this effect was lost with antibiotic administration. *Firmicutes* was significantly higher in the 20% protein defined nutrient diet compared with either the traditional diet or 2% protein defined diet (p<0.05), however there were no significant differences between groups receiving antibiotics. *Enterobacteriaceae* was higher in cecal mucosa in the traditional diet receiving group with antibiotic treatment compared with either the 20% defined diet group (p<0.01) or the 2% defined diet group (p<0.05). Total bacteria (16S) was higher in the traditional diet compared with the 2% protein diet before antibiotics (p<0.05). Total bacteria was also higher in the traditional diet group receiving antibiotics compared with either defined diet group (p<0.01).
(EPS)

S1 File 2% Protein Defined Diet Ingredient List. (PDF)

S2 File 20% Protein Defined Diet Ingredient List. (PDF)

S3 File Traditional Diet Ingredient List. (PDF)

S4 File RBD and RBD Control Diet Ingredient List. (PDF)

## References

[1] Hatheway CL. Toxigenic clostridia. Clinical microbiology reviews. 1990;3(1):66–98. 2404569 10.1128/cmr.3.1.66 PMC358141

[2] O'Connor JR, Johnson S, Gerding DN. Clostridium difficile infection caused by the epidemic BI/NAP1/027 strain. Gastroenterology. 2009;136(6):1913–24. 10.1053/j.gastro.2009.02.073 . 19457419

[3] Bartlett JG. Clinical practice. Antibiotic-associated diarrhea. The New England journal of medicine. 2002;346(5):334–9. 10.1056/NEJMcp011603 . 11821511

[4] Hurley BW, Nguyen CC. The spectrum of pseudomembranous enterocolitis and antibiotic-associated diarrhea. Archives of internal medicine. 2002;162(19):2177–84. . 12390059 10.1001/archinte.162.19.2177

[5] Cohen SH, Gerding DN, Johnson S, Kelly CP, Loo VG, McDonald LC, et al Clinical practice guidelines for Clostridium difficile infection in adults: 2010 update by the society for healthcare epidemiology of America (SHEA) and the infectious diseases society of America (IDSA). Infection control and hospital epidemiology: the official journal of the Society of Hospital Epidemiologists of America. 2010;31(5):431–55. 10.1086/651706 . 20307191

[6] Gilca R, Hubert B, Fortin E, Gaulin C, Dionne M. Epidemiological patterns and hospital characteristics associated with increased incidence of Clostridium difficile infection in Quebec, Canada, 1998–2006. Infection control and hospital epidemiology: the official journal of the Society of Hospital Epidemiologists of America. 2010;31(9):939–47. 10.1086/655463 . 20677973

[7] Kelly CP, Pothoulakis C, LaMont JT. Clostridium difficile colitis. The New England journal of medicine. 1994;330(4):257–62. 10.1056/NEJM199401273300406 . 8043060

[8] Vollaard EJ, Clasener HA. Colonization resistance. Antimicrobial agents and chemotherapy. 1994;38(3):409–14. 8203832 10.1128/aac.38.3.409 PMC284472

[9] Wilson KH. The microecology of Clostridium difficile. Clinical infectious diseases: an official publication of the Infectious Diseases Society of America. 1993;16 Suppl 4:S214–8. . 8324122 10.1093/clinids/16.supplement_4.s214

[10] Young VB, Schmidt TM. Antibiotic-associated diarrhea accompanied by large-scale alterations in the composition of the fecal microbiota. Journal of clinical microbiology. 2004;42(3):1203–6. 15004076 10.1128/JCM.42.3.1203-1206.2004 PMC356823

[11] Karlstrom O, Fryklund B, Tullus K, Burman LG. A prospective nationwide study of Clostridium difficile-associated diarrhea in Sweden. The Swedish C. difficile Study Group. Clinical infectious diseases: an official publication of the Infectious Diseases Society of America. 1998;26(1):141–5. . 9455523 10.1086/516277

[12] Pepin J, Saheb N, Coulombe MA, Alary ME, Corriveau MP, Authier S, et al Emergence of fluoroquinolones as the predominant risk factor for Clostridium difficile-associated diarrhea: a cohort study during an epidemic in Quebec. Clinical infectious diseases: an official publication of the Infectious Diseases Society of America. 2005;41(9):1254–60. 10.1086/496986 . 16206099

[13] Samore MH, Venkataraman L, DeGirolami PC, Arbeit RD, Karchmer AW. Clinical and molecular epidemiology of sporadic and clustered cases of nosocomial Clostridium difficile diarrhea. The American journal of medicine. 1996;100(1):32–40. . 8579084 10.1016/s0002-9343(96)90008-x

[14] Guerrant RL, Oria RB, Moore SR, Oria MO, Lima AA. Malnutrition as an enteric infectious disease with long-term effects on child development. Nutrition reviews. 2008;66(9):487–505. 10.1111/j.1753-4887.2008.00082.x 18752473 PMC2562291

[15] Bolick DT, Roche JK, Hontecillas R, Bassaganya-Riera J, Nataro JP, Guerrant RL. Enteroaggregative Escherichia coli strain in a novel weaned mouse model: exacerbation by malnutrition, biofilm as a virulence factor and treatment by nitazoxanide. Journal of medical microbiology. 2013;62(Pt 6):896–905. 10.1099/jmm.0.046300-0 23475903 PMC3709553

[16] Costa LB, JohnBull EA, Reeves JT, Sevilleja JE, Freire RS, Hoffman PS, et al Cryptosporidium-malnutrition interactions: mucosal disruption, cytokines, and TLR signaling in a weaned murine model. The Journal of parasitology. 2011;97(6):1113–20. 10.1645/GE-2848.1 21711105 PMC3247658

[17] Bartelt LA, Roche J, Kolling G, Bolick D, Noronha F, Naylor C, et al Persistent G. lamblia impairs growth in a murine malnutrition model. The Journal of clinical investigation. 2013;123(6):2672–84. 10.1172/JCI67294 23728173 PMC3668820

[18] David LA, Maurice CF, Carmody RN, Gootenberg DB, Button JE, Wolfe BE, et al Diet rapidly and reproducibly alters the human gut microbiome. Nature. 2014;505(7484):559–63. 10.1038/nature12820 24336217 PMC3957428

[19] Devkota S, Wang Y, Musch MW, Leone V, Fehlner-Peach H, Nadimpalli A, et al Dietary-fat-induced taurocholic acid promotes pathobiont expansion and colitis in Il10-/- mice. Nature. 2012;487(7405):104–8. 10.1038/nature11225 22722865 PMC3393783

[20] Gkouskou KK, Deligianni C, Tsatsanis C, Eliopoulos AG. The gut microbiota in mouse models of inflammatory bowel disease. Frontiers in cellular and infection microbiology. 2014;4:28 10.3389/fcimb.2014.00028 24616886 PMC3937555

[21] Teodosio NR, Lago ES, Romani SA, Guedes RC. A regional basic diet from northeast Brazil as a dietary model of experimental malnutrition. Archivos latinoamericanos de nutricion. 1990;40(4):533–47. . 2136514

[22] Warren CA, van Opstal E, Ballard TE, Kennedy A, Wang X, Riggins M, et al Amixicile, a novel inhibitor of pyruvate: ferredoxin oxidoreductase, shows efficacy against Clostridium difficile in a mouse infection model. Antimicrobial agents and chemotherapy. 2012;56(8):4103–11. 10.1128/AAC.00360-12 22585229 PMC3421617

[23] Warren CA, van Opstal EJ, Riggins MS, Li Y, Moore JH, Kolling GL, et al Vancomycin treatment's association with delayed intestinal tissue injury, clostridial overgrowth, and recurrence of Clostridium difficile infection in mice. Antimicrobial agents and chemotherapy. 2013;57(2):689–96. 10.1128/AAC.00877-12 23147742 PMC3553708

[24] Wu GD, Chen J, Hoffmann C, Bittinger K, Chen YY, Keilbaugh SA, et al Linking long-term dietary patterns with gut microbial enterotypes. Science. 2011;334(6052):105–8. 10.1126/science.1208344 21885731 PMC3368382

[25] Chen X, Katchar K, Goldsmith JD, Nanthakumar N, Cheknis A, Gerding DN, et al A mouse model of Clostridium difficile-associated disease. Gastroenterology. 2008;135(6):1984–92. 10.1053/j.gastro.2008.09.002 . 18848941

[26] Houser BA, Hattel AL, Jayarao BM. Real-time multiplex polymerase chain reaction assay for rapid detection of Clostridium difficile toxin-encoding strains. Foodborne pathogens and disease. 2010;7(6):719–26. 10.1089/fpd.2009.0483 . 20113206

[27] Lamouse-Smith ES, Tzeng A, Starnbach MN. The intestinal flora is required to support antibody responses to systemic immunization in infant and germ free mice. PloS one. 2011;6(11):e27662 10.1371/journal.pone.0027662 22114681 PMC3219679

[28] Pultz NJ, Donskey CJ. Effect of antibiotic treatment on growth of and toxin production by Clostridium difficile in the cecal contents of mice. Antimicrobial agents and chemotherapy. 2005;49(8):3529–32. 10.1128/AAC.49.8.3529-3532.2005 16048976 PMC1196291

[29] Antonopoulos DA, Huse SM, Morrison HG, Schmidt TM, Sogin ML, Young VB. Reproducible community dynamics of the gastrointestinal microbiota following antibiotic perturbation. Infection and immunity. 2009;77(6):2367–75. 10.1128/IAI.01520-08 19307217 PMC2687343

[30] Mariat D, Firmesse O, Levenez F, Guimaraes V, Sokol H, Dore J, et al The Firmicutes/Bacteroidetes ratio of the human microbiota changes with age. BMC microbiology. 2009;9:123 10.1186/1471-2180-9-123 19508720 PMC2702274

[31] Ley RE, Turnbaugh PJ, Klein S, Gordon JI. Microbial ecology: human gut microbes associated with obesity. Nature. 2006;444(7122):1022–3. 10.1038/4441022a . 17183309

[32] Panda S, El khader I, Casellas F, Lopez Vivancos J, Garcia Cors M, Santiago A, et al Short-term effect of antibiotics on human gut microbiota. PloS one. 2014;9(4):e95476 10.1371/journal.pone.0095476 24748167 PMC3991704

[33] Ling Z, Liu X, Jia X, Cheng Y, Luo Y, Yuan L, et al Impacts of infection with different toxigenic Clostridium difficile strains on faecal microbiota in children. Scientific reports. 2014;4:7485 10.1038/srep07485 25501371 PMC4265774

[34] Hussein HS, Campbell JM, Bauer LL, Fahey GC, Hogarth AJ, Wolf BW, et al Selected fructooligosaccharide composition of pet-food ingredients. The Journal of nutrition. 1998;128(12 Suppl):2803S–5S. . 9868272 10.1093/jn/128.12.2803S

[35] Hopkins MJ, Macfarlane GT. Nondigestible oligosaccharides enhance bacterial colonization resistance against Clostridium difficile in vitro. Applied and environmental microbiology. 2003;69(4):1920–7. 12676665 10.1128/AEM.69.4.1920-1927.2003 PMC154806

[36] Gaskins HR, Mackie RI, May T, Garleb KA. Dietary Fructo-oligosaccharide Modulates Large Intestinal Inflammatory Responses to Clostridium difficile in Antibiotic-compromised Mice. Microbial Ecology in Health and Disease. 2006;9(4). Epub 2011-07-21.

[37] Bosscher D, Breynaert A, Pieters L, Hermans N. Food-based strategies to modulate the composition of the intestinal microbiota and their associated health effects. Journal of physiology and pharmacology: an official journal of the Polish Physiological Society. 2009;60 Suppl 6:5–11. . 20224145

[38] Ikeda D, Karasawa T, Yamakawa K, Tanaka R, Namiki M, Nakamura S. Effect of isoleucine on toxin production by Clostridium difficile in a defined medium. Zentralblatt fur Bakteriologie: international journal of medical microbiology. 1998;287(4):375–86. . 9638867 10.1016/s0934-8840(98)80174-6

[39] Baxter R, Ray GT, Fireman BH. Case-control study of antibiotic use and subsequent Clostridium difficile-associated diarrhea in hospitalized patients. Infection control and hospital epidemiology: the official journal of the Society of Hospital Epidemiologists of America. 2008;29(1):44–50. 10.1086/524320 . 18171186

[40] Belkaid Y, Hand TW. Role of the microbiota in immunity and inflammation. Cell. 2014;157(1):121–41. 10.1016/j.cell.2014.03.011 24679531 PMC4056765

[41] Solomon K. The host immune response to Clostridium difficile infection. Therapeutic advances in infectious disease. 2013;1(1):19–35. 10.1177/2049936112472173 25165542 PMC4040718

[42] Bolick DT, Kolling GL, Moore JH 2nd, de Oliveira LA, Tung K, Philipson C, et al Zinc deficiency alters host response and pathogen virulence in a mouse model of enteroaggregative escherichia coli-induced diarrhea. Gut microbes. 2014;5(5):618–27. 10.4161/19490976.2014.969642 . 25483331 PMC4615194

