# Correction: Defined Nutrient Diets Alter Susceptibility to *Clostridium difficile* Associated Disease in a Murine Model

**DOI:** 10.1371/journal.pone.0137037

**Published:** 2015-09-15

**Authors:** John H. Moore, Caio C. D. Pinheiro, Edna I. Zaenker, David T. Bolick, Glynis L. Kolling, Edward van Opstal, Francisco J. D. Noronha, Pedro H. Q. S. De Medeiros, Raphael S. Rodriguez, Aldo A. Lima, Richard L. Guerrant, Cirle A. Warren

There are errors in the Funding section. The correct funding information is as follows: Funding was provided in part by NIH/NIAID U01 AI075526 and in part by the Bill & Melinda Gates Foundation OPP1066140. The funders had no role in study design, data collection and analysis, decision to publish, or preparation of the manuscript.
