# Supplementary material for: Defined Nutrient Diets Alter Susceptibility to Clostridium difficile Associated Disease in a Murine Model
Source: PLoS One. 2015 Jul 16;10(7):e0131829. doi: 10.1371/journal.pone.0131829 (PMC4504475; doi:10.1371/journal.pone.0131829)
Supplement: S2 File — (PDF) [file pone.0131829.s005.pdf]

TD.08678 20% Protein Diet

Formula	g/Kg
Casein	**230.0**
DL-Methionine	**3.0**
Sucrose	**150.0**
Corn Starch	**349.56**
Maltodextrin	**120.0**
Corn Oil	**52.3**
Cellulose	**50.0**
Vitamin Mix, Teklad (40060)	**10.0**
Mineral Mix, Ca-P Deficient (79055)	**13.37**
Calcium Phosphate, dibasic	**16.66**
Calcium Carbonate	**5.1**
Ethoxyquin, antioxidant	**0.01**

Footnote

A 20% protein diet with 15% sucrose. Modified from TD.91352 (protein adjusted series) to reduce sucrose and increase starch.

Selected Nutrient Information[1]

	% by weight	% kcal from
**Protein**	20.3	22.3
**Carbohydrate**	58.3	64.1
**Fat**	5.5	13.6
**Kcal/g**	**3.6**	

[1] Values are calculated from ingredient analysis or manufacturer data

Teklad Diets are designed & manufactured for research purposes only.

Speak With A Nutritionist

- **(800) 483·5523**
- **askanutritionist@harlan.com**

Harlan Laboratories · PO Box 44220 · Madison, WI 53744-4220

www.harlan.com

Key Features

- **Purified Diet**
- **Normal Protein (Control)**
- **Rodent**

Key Planning Information

- *Products are made fresh to order*
- Store product at 4°C or lower
- Use within 6 months (applicable to most diets)
- Box labeled with product name, manufacturing date, and lot number
- Replace diet at minimum once per week

  More frequent replacement may be advised
- Lead time:
  - 2 weeks non-irradiated
  - 4 weeks irradiated

Product Specific Information

- 1/2" Pellet or Powder (free flowing)
- Minimum order 3 Kg
- Irradiation available upon request

Options (Fees Will Apply)

- Rush order (pending availability)
- Irradiation (see Product Specific Information)
- Vacuum packaging (1 and 2 Kg)

International Inquiry

·Outside U.S.A. or Canada·

- **askanutritionist@harlan.com**

Place Your Order (U.S.A. & Canada)

· Place Order · Obtain Pricing ·
· Check Order Status ·

- **(800) 483·5523**
- **(608) 277·2066** *facsimile*
- **tekladinfo@harlan.com**

Helping you do research better

Harlan, Harlan Laboratories, Helping you do research better,
and the Harlan logo are trademarks and trade names of Harlan Laboratories, Inc.
© 2008 Harlan Laboratories, Inc.
