# Supplementary material for: Defined Nutrient Diets Alter Susceptibility to Clostridium difficile Associated Disease in a Murine Model
Source: PLoS One. 2015 Jul 16;10(7):e0131829. doi: 10.1371/journal.pone.0131829 (PMC4504475; doi:10.1371/journal.pone.0131829)
Supplement: S4 File — (PDF) [file pone.0131829.s007.pdf]

D09051102 and D09081701

OpenStandard Diet With 20 kcal% Protein, 15 kcal% Fat and 65 kcal% Carbohydrate and Same with Reduced Protein, Calcium, Potassium, Sodium, Chloride, and Phosphorus

**Product #**	**D09051102**		**D09081701**	
	Control Diet		*Deficient Diet*	
	gm%	*kcal%*	gm%	*kcal%*
Protein	19.0	*20*	6.5	*7*
Carbohydrate	63.1	*65*	81.9	*88*
Fat	6.5	*15*	2.1	*5*
Total		*100*		*100*
kcal/gm	3.77		3.77	
**Ingredient**	**gm**	***kcal***	**gm**	***kcal***
Casein	200	*800*	71	*284*
L-Cystine	3	*12*	1.07	*4*
Corn Starch	346	*1384*	557	*2228*
Maltodextrin 10	45	*180*	70	*280*
Dextrose	250	*1000*	250	*1000*
Sucrose	0	*0*	2.41	*10*
Cellulose, BW200	75	*0*	75	*0*
Inulin	25	*25*	25	*25*
Soybean Oil	70	*630*	23.3	*210*
Mineral Mix S10026	10	*0*	0	*0*
Mineral Mix S10026A	0	*0*	5	*0*
Dicalcium Phosphate	13	*0*	11	*0*
Calcium Carbonate	5.5	*0*	0	*0*
Potassium Citrate, 1 H2O	16.5	*0*	8.2	*0*
Sodium Chloride	0	*0*	1.9	*0*
Vitamin Mix V10001	10	*40*	10	*40*
Choline Bitartrate	2	*0*	2	*0*
Red Dye #40, FD&C	0	*0*	0.05	*0*
Blue Dye #1, FD&C	0.025	*0*	0	*0*
Yellow Dye #5, FD&C	0.025	*0*	0	*0*
**Total**	**1071.05**	***4071***	**1112.93**	***4081***

Formulated by Research Diets, Inc.

8/24/09
